# Graphene Hybrid Metasurfaces for Mid-Infrared Molecular Sensors

**DOI:** 10.3390/nano13142113

**Published:** 2023-07-20

**Authors:** Tom Yager, George Chikvaidze, Qin Wang, Ying Fu

**Affiliations:** 1 Institute of Solid State Physics, University of Latvia, LV-1063 Riga, Latvia; georg.chikvaidze@cfi.lu.lv; 2 RISE Research Institutes of Sweden AB, Box 1070, SE-164 25 Kista, Sweden; qin.wang@ri.se; 3 School of Information Technology, Halmstad University, SE-301 18 Halmstad, Sweden

**Keywords:** graphene, mid-infrared, metasurface, gas sensor, photodetector

## Abstract

We integrated graphene with asymmetric metal metasurfaces and optimised the geometry dependent photoresponse towards optoelectronic molecular sensor devices. Through careful tuning and characterisation, combining finite-difference time-domain simulations, electron-beam lithography-based nanofabrication, and micro-Fourier transform infrared spectroscopy, we achieved precise control over the mid-infrared peak response wavelengths, transmittance, and reflectance. Our methods enabled simple, reproducible and targeted mid-infrared molecular sensing over a wide range of geometrical parameters. With ultimate minimization potential down to atomic thicknesses and a diverse range of complimentary nanomaterial combinations, we anticipate a high impact potential of these technologies for environmental monitoring, threat detection, and point of care diagnostics.

## 1. Introduction

Distinctive mid-infrared (MIR) molecular vibrations, ranging between ~2 and 12 μm, act as characteristic ‘molecular fingerprints’ for label-free identification of a wide range of chemicals and biomolecules. Usefully, atmospheric transparency windows, at 3–5 μm and 8–13 μm, enable a range of applications such as CO_2_ gas sensing and alcohol detection. Within the same MIR spectral range, black body radiation can also be utilised for photodetection and thermal imaging technologies. Taken together, MIR technologies have a substantial role in environmental monitoring, medical diagnosis, and security. However, in comparison to other wavelength regions, there exists a relative lack of MIR sources, detectors, and methodologies.

The current state-of-the-art technologies for MIR sensors are based on semiconductor bulk or quantum structures, such as gallium arsenide (GaAs), indium antimonide (InSb), and mercury-cadmium-telluride (MCT) [1]. Whilst these detectors offer very high sensitivity performance, their wider implementation and dissemination is significantly limited by a combination of high costs, limited spectral range, scarce materials, temperature sensitivity, and need for cooling. As a result, commercially available MIR sensing technologies are typically bulky and expensive, requiring specially controlled operating conditions. Whilst MIR technologies continue to develop, alternative materials and approaches are in high demand to meet the multiple challenges of device sensitivity, spectral range, size, cost, and power consumption [1,2,3].

A new family of low dimensional nanomaterials (graphene, transition metal dichalcogenides, topological insulators) offer unique optoelectronic functionalities and new technological solutions beyond those attainable with conventional semiconductors [4,5,6,7,8,9]. Graphene-based devices, in particular, attracted extensive research and attention due to their unique optoelectronic properties, broadband absorption, and electronic tuneability. For example, graphene shows good potential as atomically thin transparent conductive electrodes, combining high optical transparency (over 97%) for a wide range of wavelengths with high charge carrier mobilities for ultrafast devices [10,11]. Device performances can be extended down to very low charge carrier densities and are robust over a broad range of temperatures, allowing relatively relaxed operational requirements and conditions [12,13,14].

One practical limitation is the effect of charge inhomogeneity and scattering arising from the underlying substrate (typically SiO_2_, SiC, quartz) or from environmental and atmospheric dopants [15,16,17,18]. A range of technological solutions is being developed for surface control and stabilisation, such as encapsulation by polymers [19,20] or boron nitride layers [21,22]. In addition, improvements to the growth and transfer processes allow integration of graphene layers to a wide range of substrates and devices, with increasingly demonstrated and established CMOS compatibility [23,24]. For the optimisation of these optoelectronic devices, it is important to understand the material properties and performance, both individually and in combination, in the operational range of interest (wavelength, atmosphere, temperature). However, to date, the majority of literature reports focused on the visible to UV wavelengths, whilst the mid-infrared spectral region remained relatively underexplored.

A significant challenge for these nanoscale materials is to achieve sufficiently strong optical coupling, due in part to a size mismatch of several orders of magnitude with micrometre scale wavelengths. One promising approach to increase the photonic interaction with atomic materials, and molecules, is through nanoplasmonics [25,26]. Graphene is in itself capable of exhibiting plasmonic behaviour that can be enhanced and tuned by geometric patterning [27,28,29]. An alternate approach is to combine graphene with other plasmonic materials to form hybrid plasmonic structures, through near-field coupling [30,31,32,33]. Taking this approach one step further, the plasmonic nanoantenna can also be precision designed and patterned to form sub-wavelength arrays of quasi-2D metastructures or metasurfaces [34,35,36,37].

Metasurface technologies have a high complementarity and compatibility with 2D nanomaterials, with integration potential greater than the sum of their parts. One important benefit is precision photonic design of spectral selectivity, polarization, and focussing based primarily on lateral geometrical patterning where underlying fabrication methods are already well developed and CMOS compatible. Lateral geometric selectivity also allows for relatively straightforward multiplexing or pixelation of different devices, for enhanced selectivity, comparative analysis, and multi- or hyper-spectral detection and imaging. From the reverse perspective, functional 2D nanomaterials, such as graphene, offer additional functionalities such as dynamic tunability of the spectral photoresponse wavelength by electrostatic or electrochemical gating [38,39,40].

In recent years, there have been several significant reports exploring such MIR hybrid-graphene metasurfaces with potential for molecular sensing applications [32]. There can be considered three primary molecular sensing mechanisms for such systems: (i) optical sensing by photodetection, (ii) enhancement of the molecular absorption, (iii) peak wavelength shifts and modulation. For example, proof-of-principle photodetection devices were demonstrated by graphene-metal square micropatch arrays with MIR wavelength selective photoresponse [41,42] and ultrafast broadband photodetection by gold nanostripes with MIR photodetector responsivity up to ~2 A.W^−1^ at room temperature [43]. However, substantial challenges remain in the design, and a demonstration of these technologies to combine high photoresponsivity, precisely targeted wavelength selectivity, and implementation under application relevant operating conditions.

In this work, we demonstrated the design, fabrication, and characterization of hybrid-graphene metasurface devices towards geometrically tuneable molecular sensors for CO_2_ and alcohol detection. Electron beam lithography-based nanofabrication was combined with micro-Fourier transform infrared (μFTIR) spectroscopy to understand the key geometric tuning parameters for optimising MIR interaction and wavelength selectivity. The role of graphene for optoelectronic hybrid metasurface devices was confirmed and clarified by finite-difference time-domain (FDTD) studies. Moreover, the technological approaches described are readily scalable for industrial applications, where there is a high demand in a wide range of applications such as air quality inspection, automotive vehicle safety systems, alcohol sensing, healthcare, and security.

## 2. Materials and Methods

### 2.1. Metal Metasurface Fabrication

Asymmetric metal nanoantenna metasurface arrays were fabricated by electron beam lithography and optically characterised by micro-Fourier transform infrared spectroscopy. A SiO_2_ surface, thickness 192 nm, was thermally grown by standard PECVD on a commercially available double-side-polished silicon wafer, of thickness 525 μm (MicroChemicals, Ulm, Germany). The wafer was diced into chips of dimensions 10 × 10 or 20 × 20 mm^2^. The nanostructured arrays were then defined with a Raith eLine Plus electron beam lithography system (Raith, Dortmund, Germany) using polymethyl methacrylate (950PMMA A4 PMMA, Micro Resist Technology, Berlin, Germany) spin-coated at 4000 r.p.m. for 1 min, baked at 180 °C. Initial geometrical design parameters were determined with input from FDTD studies. The metal nanostructures were deposited in an Edwards Auto 306 thermal evaporator (Edwards, Burgess Hill, UK) (vacuum pressure 10^−6^ mBar), to cover large surface areas (2 × 2 to 10 × 10 mm^2^) of the SiO_2_ substrates. Following metal lift-off and solvent based cleaning (acetone, isopropanol), the surface geometries were analysed by scanning electron microscopy (SEM), using the same eLine system.

Dimensions of the metal surface arrays were determined with an approximate measurement uncertainty of 0.025 μm, originating from geometric non-uniformity of fabricated metal nanoantenna and SEM measurement precision. Both the array-to-array and wafer-to-wafer repeatability was found to be within this same uncertainty range. Deposited metal thicknesses were verified with a Dektak 150 Surface Profiler (Veeco, Santa Barbara, CA, USA) within 2 nm. Thicknesses of the deposited Au nanoantenna structures were 50 ± 5 nm.

### 2.2. Integration of Graphene with Metal Arrays to Form Hybrid Metasurfaces

Two approaches were implemented for integrating the metal metastructures with monolayer CVD graphene (Graphenea, San Sebastián, Spain): (1) graphene transfer onto pre-fabricated and characterised metal arrays and (2) direct metal deposition on the graphene surface. Transferring graphene allowed direct comparison of optically characterized surfaces and reduced risks related to graphene–metal adhesion. Nominally single layer graphene was acquired from and transferred on to the fabricated and optically characterised metal arrays on SiO_2_ substrates by an adhesive transfer method [24]. The presence of graphene layers was confirmed by a combination of contrast enhanced optical microscopy [44], electrical conductivity, and confocal micro-Raman analysis (S&I Spectroscopy & Imaging, Warstein, Germany).

Graphene optoelectronic device structures were patterned by electron beam lithography, graphene plasma ashing, metallisation, and lift-off as described in [45]. Material and device conductivity were measured at room temperature in an electronic probe station and 2450 SourceMeter (Keithley, Cleveland, OH, USA). In the absence of graphene, macroscopic surface conductivity was too low to measure for the Au metasurfaces on SiO_2_, with resistivities of a few Ω on the Au contacts, indicating correctly isolated Au nanostructures.

With the addition of graphene, two-dimensional sheet resistivities of the devices were estimated as ~0.7 ± 0.3 kΩ/square. By considering commercial material standardisation and comparing to previous characterisation data, the graphene device mobilities were estimated as ~1000 cm^2^·V^−1^·s^−1^, with two dimensional charge carrier densities of ~1 × 10^12^ cm^−2^. At this measured charge carrier density, significant changes or asymmetry were not observed by comparing the resistivities of the asymmetric graphene–metal metastructures, suggesting that graphene conductivity dominates the device conductivity in the measured range.

### 2.3. Fourier-Transform Infrared Characterisation of the Metasurfaces

Micro reflection and transmission Fourier-transform infrared spectroscopy (FTIR) measurements were performed by a VERTEX 80v FT-IR spectrometer attached to a HYPERION 2000 FTIR microscope (Bruker, Ettlingen, Germany), which allowed to measure both transmission and reflection spectra of the samples. The FTIR spectra were recorded in the range of 5000–500 cm^−1^ (2–20 μm), with a resolution of 2 cm^−1^, measured over areas from 40 × 40 μm^2^ to 1 × 1 cm^2^. Polarization measurements used an additional model P03 IR Polarizer (Bruker, Ettlingen, Germany).

### 2.4. Finite-Difference Time-Domain Studies

The applied FDTD code was developed in-house, as described in several previous studies [46,47,48]. The purpose of the development and application of our own FDTD codes was to tackle the considerable difference between the mid-infrared wavelengths (2~10 μm) of interest and the thickness (*d* = 0.335 nm) of the graphene sheet. Dielectric coefficients of gold were numerically described using Lorentz equation with two poles by fitting refractive index and extinction coefficient data obtained from [49]. Periodic boundary conditions were applied in the *x* and *y* directions, while the ends of the calculation domain in the *z* dimension were simulated by perfectly matched layers (PMLs). Time step duration in the FDTD calculations was 1.83 × 10^−13^ s, while a mesh size of 0.012 nm was used, so that detailed features of the electromagnetic wave within the single graphene sheet were properly resolved. The total number of simulated time steps was 16 × 10^5^; it was sufficient that all fields propagated away from the calculation domain of the FDTD study. The FDTD code required up to 200 GB RAM in a mini-supercomputer Intel(R) Xeon(R) 144 cores, 500 GB RAM, 20 TB HDD.

## 3. Results 

### 3.1. Geometric Tuneability of Metal Metasurfaces on SiO_2_

Metasurfaces, comprised of asymmetric metal nanoantenna arrays, were initially designed based on FDTD studies, which indicated strong interactions with electromagnetic waves, even for sparse metal arrays, with significantly enhanced reflectance (85%), a substantial diffraction (10%), and a much-reduced transmittance (5%) for an array of only 15% surface metal coverage [48]. Importantly, the propagating electromagnetic fields were estimated to be transiently concentrated around the surface nanolayer (e.g., graphene) in a time duration on the order of tens of nanoseconds, suggesting a novel efficient near-field optical coupling.

**Figure 1:**
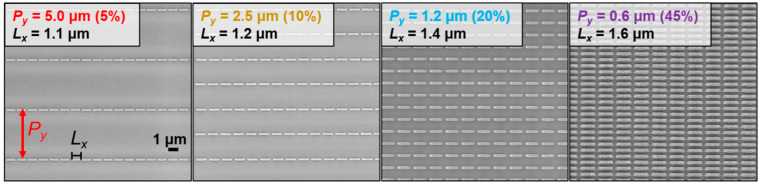
Scanning electron microscopy images of the fabricated metal metasurfaces patterned on SiO_2_ covered substrates. Metal thickness (50 nm), design width (200 nm), and nanogap distance (200 nm) were fixed, whilst the lateral pitch *P*_y_ and horizontal metal lengths *L*_x_ were varied. From left to right, the density of metal antennas and coverage % are increased, whilst the horizontal metal lengths *L*_x_ were also slightly increased to compensate for *P*_y_-induced blueshifts and targeting CO_2_ (4.25 μm) with their peak photoresponse wavelengths.

Since the direct interaction of unpatterned graphene in the midinfrared can be relatively weak, in this approach, we began with fabrication and analysis of metal nanoantenna metasurface on SiO_2_-Si substrates, followed by description and analysis of the integrated hybrid graphene metasurfaces. Figure 1 shows the geometry of one of the studied metastructure arrays measured by scanning electron microscopy. For all structures presented in this study, the designed metal thickness (*L_z_* = 50 nm ≈ *λ_MIR_*/80), width (*L_y_* = 200 nm), and nanogap lengths (*g_x_* = *P_x_* − *L_x_* = 200 nm) remained fixed, whilst the metal length (*L*_x_) and lateral pitch (*P*_y_) were varied. Metal coverages were estimated as *M*_%_ = (*L_x_* × *L_y_*)/(*P_x_* × *P_y_*).

**Figure 2:**
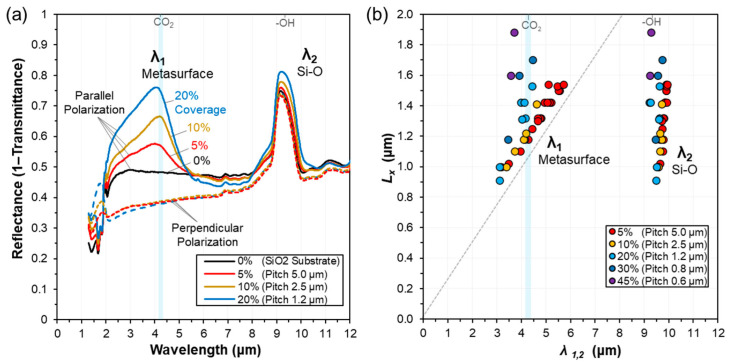
(**a**) Linear polarized micro-FTIR spectra of the fabricated metasurfaces shows two clear mid-infrared photoresponses peaks, λ_1_, λ_2_. Here, the metasurface photoresponses λ_1_ were targeted at the 4.25 μm absorption peak for CO_2_, with wavelength dependent peak interaction intensities increasing with the metal coverage. (**b**) Measured dependence of the peak-response wavelengths (λ_1_, λ_2_) on the designed metal length (*L*_x_), for different array pitch (*P*_y_). λ_1_ can be attributed to the metal metasurface geometry and increases with metal length *L*_x_ whilst decreasing with sub-wavelength metal coverage densities (Pitch, %Metal). The dashed line follows the expected trend for isolated nanoantenna, derived in Section 3.3.

The MIR photoresponse of the fabricated metal metasurfaces was investigated by micro-FTIR spectroscopy. Figure 2a shows FTIR spectra comparing metasurfaces with widely varying metal coverages, defined by the lateral pitch *P*_y_. Two main transmittance minima were observed, defined here as *λ*_1_ and *λ*_2_. The second transmittance minima, around 9–10 μm (*λ*_2_), was also observed for unpatterned reference regions of Si–SiO_2_ (SiO_2_ layer thickness 192 nm), which was attributed to asymmetric Si–O stretching modes for the sample substrate [50]. However, the interaction strength appeared locally enhanced with increasing coverage density of the metasurface arrays. In contrast, the transmittance minimum, *λ*_1_, displayed around 4 μm, was absent for the unpatterned Si–SiO_2_ regions. This minimum depended strongly on the IR polarization and metal geometry, and can be fully attributed to the patterned metasurfaces. Peak interaction strengths (transmittance minima, reflectance maxima) were observed to increase with the metal coverage in the range of 3% (*P*_y_ = 10 μm) − 20% (*P*_y_ = 1.2 μm). For higher density arrays of nanoantenna, the intensity increases appeared to saturate, with a slight broadening of the peak.

The peak response wavelengths of the main MIR features were investigated as a function of the metasurface geometry. Figure 2b displays FTIR analysis of the peak mid-IR photoresponse for 38 unique fabricated and characterised metal metasurface arrays on SiO_2_ substrates. Both *λ*_1_ and *λ*_2_ displayed geometry dependant tuneability. It was found that the primary geometric variable was the nanoantenna metal length *L_x_*, whilst an important perturbation was induced by the lateral pitch *P_y_*. The shorter infrared peak wavelength response *λ*_1_ exhibited the strongest dependence on the array geometries. As with *λ*_1_, transmittance minimum *λ*_2_ was also observed to shift with varying metal length and lateral pitch (*L_x_*, *P_y_*). However, the shift in *λ*_2_ was significantly less sensitive to changes in the geometry, only ranging between ~9.2 and 10.0 μm for the fabricated structures. The smaller observed blueshift in λ_2_, at high metal coverage, was understood to be the effect of the non-symmetric density-of-states of the Si–O vibration modes in the three-dimensional SiO_2_ layer (thickness 192 nm) [51,52]. The enhanced transmittance at *λ*_2_ was the result of the transiently concentrated electromagnetic field around the surface nanolayer which strongly activates the Si–O vibration modes in the SiO_2_ layer. Midinfrared photoresponses were qualitatively similar for both peaks from room temperature down to 10 Kelvin, suggesting temperature resilient performance, however with an apparent blueshift of approximately 150 nm at low temperatures.

The characterised photoresponse for both features were also observed to be less critically sensitive to other geometric factors, varied within ~50%, such as metal thickness, metal width, longitudinal pitch, nanogaps, and SiO_2_ dielectric thickness. Combining this information, enabled reliable reproduction of metasurfaces with optimised and well-defined photoresponse for integration with graphene, and towards devices for targeted molecular sensing applications.

### 3.2. Graphene Metasurface Device Integration and Photoresponse

**Figure 3:**
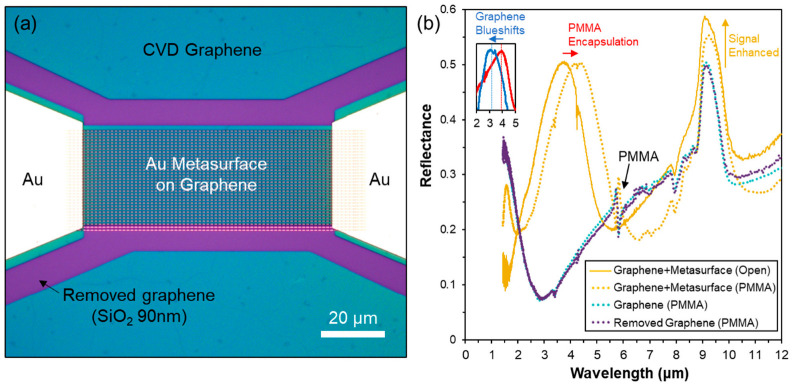
(**a**) Example optoelectronic hybrid graphene metasurface device patterned on a monolayer CVD graphene—SiO_2_ substrate, imaged by LUT contrast enhanced optical microscopy during processing [44]. (**b**) Micro-FTIR reflectance comparing the different material related photoresponse of different hybrid graphene metasurface optoelectronic device areas, measured in ambient conditions. The figure inset shows a blueshifted photoresponse peak with the addition of graphene, as measured for the same geometrical metastructures.

Two technological approaches were investigated to integrate graphene with the geometrically optimised metal arrays to form hybrid metasurface devices. In the first approach, monolayer CVD graphene was transferred onto the pre-characterised SiO_2_–metal metasurfaces, enabling direct comparison of the photoresponse [24]. Alternatively, the nanofabrication of the metasurfaces was replicated on substrates where graphene already covered the full surface (Figure 3a), allowing simplified and reproducible device processing [45]. With the addition of graphene layers, FTIR spectra were qualitatively similar to uncovered metal arrays on bare SiO_2_, with the persistent presence of λ_1_ and λ_2_ (Figure 3b). However, with the addition of graphene monolayers, an additional blueshift was observed at λ_1_ for each of the measured array structures (Table 1). Significant signal enhancements were observed, of Si–O (at 9.5 μm) and PMMA (at 5.8 μm) peaks of +16–19% and +11%, respectively. Considering that the total metal coverages were only 20%, and that the most active focus area around and between the poles of antenna elements was close to ~1% coverage, this represents an order of magnitude enhancement of the local molecular signals.

### 3.3. Time-Resolved FDTD Study of the Infrared Pulse Transmission of Graphene

To understand our experimental results, we theoretically investigated infrared pulse transmission through hybrid graphene metasurfaces by the FDTD method. The wavelength range of interest was within the midinfrared range, 2–10 μm, so the intraband conductivity of graphene sheet is adopted [27,53]:

(1) $$\sigma =\frac{ie^{2}E_{f}}{\pi \hbar^{2}\left(\omega +i/\tau \right)}$$

where $E_{f}$ is the Fermi level, in the order of approximately 0.2 eV and *τ* = 100 fs, and the real and imaginary parts of the relative dielectric coefficient are

(2) $$\varepsilon =\varepsilon \prime +i\varepsilon \prime \prime =1+\frac{i\sigma }{\varepsilon_{0}\omega d}$$

for the graphene plasmonic resonance to the IR radiation as functions of the wavelength of the IR radiation, where *d* = 0.335 nm is the thickness of the graphene sheet. Note that ε′ is negative, and the absolute value increases quickly following the increase of the IR wavelength. ε′′ increases with the IR wavelength, and is positive, indicating that the transmission of the IR waves through the graphene sheet is very lossy. Note that in Equations (1) and (2), $E_{f}$ = 0.2 eV and *τ* = 100 fs are adopted from [27,53]. In [53], the graphene mobility was 2700 cm^2^ V^−1^ s^−1^, whilst the experimental graphene properties in this study were estimated as 1000 cm^−2^/V s (at ~1 × 10^12^ cm^−2^). By scaling τ proportional to mobility, we obtain a new τ = 100/2.7 fs. Such a modification does not affect Equations (1) and (2), since 1/τ = 1.0/100.0 × 10^−15^ = 1.0 × 10^13^ s^−1^, 1/(100 × 10^−15^/2.7) = 2.7 × 10^13^ s^−1^, while in the wavelength range of interest, ω = 2π c/λ = 6.28 × 3 × 10^8^/4.0 × 10^−6^ = 4.7 × 10^14^ s^−1^ is much larger than the τ^−1^ factor.

The first theoretical study subject was to understand the almost identical FTIR spectra of graphene and removed graphene in Figure 3b. In other words, a single graphene sheet does not affect the optical properties of SiO_2_-Si. This sounds reasonable, since the wavelengths of the IR waves were much larger than the sub-nano-feature sizes of the graphene sheet (0.335 nm); so, macroscopically, the IR waves should transmit through without perceivable perturbation. However, previous studies reported significant crests and troughs in the IR transmission spectra through single graphene sheet, e.g., [54].

We started with a single graphene sheet in vacuum. The transmittance of the IR plane wave at normal incidence through this single graphene sheet extended in the *xy* plane positioned at *z* = 0 is easily calculated by applying Fresnel’s equations

(3) $$r_{12}=\frac{E_{r}}{E_{i}}=\frac{{\tilde{n}}_{1}-{\tilde{n}}_{2}}{{\tilde{n}}_{1}+{\tilde{n}}_{2}}\;\;\;,\;\;\;t_{12}=\frac{E_{t}}{E_{i}}=\frac{2{\tilde{n}}_{1}}{{\tilde{n}}_{1}+{\tilde{n}}_{2}}$$

where *E_r_*, *E_i_*, and *E_t_* denote the electric field of the reflected, incident, and transmitted wave, respectively, between medium 1 denoted by complex refractive index *ñ*_1_ and medium 2 having *ñ*_2_. Let *ñ* = *n* + *iκ* be the complex refractive index of the thin graphene film (denoted as medium 2), and the measurement is performed in air so that the refractive indices of the spaces above the upper interface of the graphene sheet (medium 1) and below the lower interface of the graphene sheet (medium 3) are 1.0. By Equation (3), the reflection and refraction coefficients at the upper and lower interfaces are

(4) $$r_{12}=\frac{1-\tilde{n}}{1+\tilde{n}}\;,\;{\;\;t}_{12}=\frac{2}{1+\tilde{n}}\;,\;{\;\;\;r}_{21}=r_{23}=\frac{\tilde{n}-1}{\tilde{n}+1}\;{,\;\;\;t}_{21}=t_{23}=\frac{2\tilde{n}}{\tilde{n}+1}$$

for a single reflection and transmission. Here, *r*_12_ is the reflection of the plane wave from medium 1 back to medium 1 reflected by the upper interface of the graphene sheet, *t*_12_ is the refraction from medium 1 into medium 2 at the upper interface. *r*_23_ and *t*_23_ are likewise defined but at the lower interface. Note that *r*_12_ = −*r*_23_. The series of the transmitted waves are

(5) $$\frac{E_{t}}{E_{i}}=e^{i\delta }t_{12}t_{23}\left(1+\beta +\beta^{2}+\cdots \right)=e^{i\delta }t_{12}t_{23}\;\underset{n\to \infty }{lim}\sum_{i=0}^{n}\beta^{i}$$

where *δ* = *ωñd*/*c*_0_ and *β* = *e^iδ^* *r*_23_ *r*_21_. It is easy to see that the result of the above infinite summation is

(6) $$\frac{E_{t}}{E_{i}}=\frac{e^{i\delta }t_{12}t_{23}}{1+e^{2i\delta }r_{12}r_{23}}$$

Since the optical power of the transmitted light is

(7) $$S_{t}=2c_{0}\varepsilon_{0}E_{t}^{2}$$

while the incident optical power is *S_i_* = 2*c*_0_*ε*_0_*E_i_*^2^ from which we obtain the transmittance *T* through the thin graphene film

(8) $$T=\frac{\left|{\left\langle S_{t}\right\rangle }_{t}\right|}{\left|{\left\langle S_{i}\right\rangle }_{t}\right|}={\left|\frac{e^{i\delta }t_{12}t_{23}}{1+e^{2i\delta }r_{12}r_{23}}\right|}^{2}.$$

**Figure 4:**
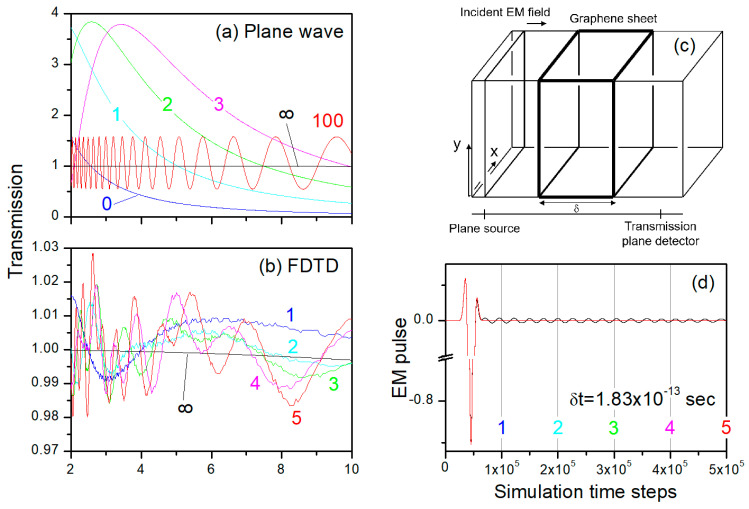
(**a**) Transmission coefficient of normal incident IR plane waves through a single graphene sheet. (**b**) Time-resolved transmission spectrum by FDTD shown in (**c**). (**d**) The *x*-component of the transmitting electromagnetic field (black line: *Ex* through the graphene sheet; red line: transmission without the graphene sheet).

The numerically calculated transmittance, by Equation (5) with limited summation over *n*, then Equation (6) for *n* = ∞ (the black line marked with “∞”) are presented in Figure 4a. For *n* = ∞, the transmittance is very close to 1.0 due to the extremely thin layer thickness of the graphene sheet (*d* = 0.335 nm) and appears featureless as a function of the IR wavelength. However, when *n* is limited, strong oscillation in the transmittance spectrum is observed.

Next, we performed FDTD numerical calculations using in-house FDTD codes [46,47,48]. Numerical results were carefully examined in both space and time domains. 

FDTD-calculated transmission spectrum of the graphene sheet is presented in Figure 4b as a function of the number of FDTD simulation steps, where the graphene sheet was placed on the *xy* plane, and the IR pulse impinged on the graphene structure along the *z* axis (see Figure 4c). As compared with the black ∞ line in Figure 4a, which is also presented in Figure 4b for direct comparison, the FDTD transmission spectrum oscillated strongly along the simulation time.

The critical aspect about results of Equation (6) and FDTD calculation is that Equation (6) is derived for a time interval of infinite length, i.e., *n* → ∞ in Equation (5). When we calculated the transmittance as a function of *n*, as a means to emulate measurements of finite time intervals, Equations (5) and (6) produced crests and troughs in the transmission spectrum (see Figure 4a), similar to the oscillations in Figure 4b. For the single graphene sheet, there existed a very substantial difference between *n* = 100 and *n* = ∞. This can be expected since the intensity of the IR wave in the graphene sheet will reduce gradually due to both the absorption (loss, *ε*′′ ≠ 0, but the real loss was negligibly small due to thin thickness) but mainly the transmission.

This also explains the time-dependence of the FDTD-calculated transmission spectrum presented in Figure 4b. The *E*_x_ field of the transmitting electromagnetic field is shown in Figure 4d, indicating that the major electromagnetic field passed through the graphene sheet already at approximately the time step of 10^5^ (the time duration of each time step is *δt* = 1.83 × 10^−13^ s in FDTD); we still observed significant EM field passing through the transmission detector, which caused the strong oscillation in the FDTD-calculated transmission spectrum in Figure 4b. This effect was very significant for the graphene sheet since the dielectric coefficient of the graphene sheet was very large, so that the effective light speed there was much slowed.

### 3.4. FDTD Analysis of the Hybrid Graphene Metasurfaces

**Figure 5:**
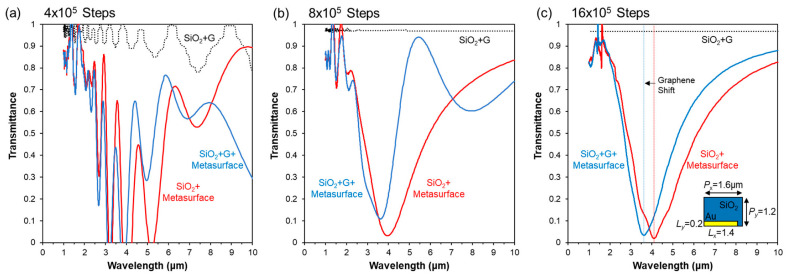
FDTD transmittance spectra of parallel (*E*_x_, *H*_y_) polarized EM fields. The gold metasurface geometry was *L_x_* × *L_y_* × *L_z_* = 1.4 × 0.2 × 0.05 μm^3^, with pitch *P_x_* × *P_y_* = 1.6 × 1.2 μm^2^. The number of simulation steps was increased from (**a**) 4 × 10^5^, (**b**) 8 × 10^5^, up to (**c**) 16 × 10^5^ to allow all transmitted EM field through the detector. The addition of graphene is associated with a blueshift in the photoresponse, in close agreement with experimental FTIR spectra (Table 1).

Here, we studied periodic gold nanoantenna metasurfaces embedded in SiO_2_ then covered with a single graphene sheet schematically shown in Figure 5. The two-dimensional metasurface array of gold nanorod antenna was placed on the surface of an insulating SiO_2_/Si substrate. The size of the metal patch was denoted as *L_x_* × *L_y_* × *L_z_*, where *L_x_* = 1.4 μm is the metal length in the *x* direction, *L_y_* = 0.2 μm is the metal width in the *y* direction, and *L_z_* = 0.05 μm is the metal thickness in the *z* direction. The periods of the array are denoted as *P_x_* = 1.6 μm and *P_y_* = 1.2 μm in the *x* and *y* direction, respectively.

Figure 5 shows the transmittance spectra of the three sub-micron structures at three different simulation times. Because of the large dielectric coefficients of both the graphene sheet and the gold nanoantenna as well as the long wavelengths of interest (1∼10 μm), the transmittance spectra shown in Figure 5a strongly oscillated because the transmission detector in the simulation was placed within the residual electromagnetic fields captured around the graphene sheet and gold nanoantenna, diffracted from the *z* propagation direction to propagate along the *x* and *y* directions, which persist a long time since the sub-micron structures are periodic along the *xy* plane. Only after ~12 × 10^5^ simulation steps, all transmitted EM field passed through the detector and the transmittance spectra converge (see Figure 5c). By comparison, here, we emphasised the technical importance of avoiding numerical artifacts for the system (Figure 5a,b).

Similar to Figure 4, the transmittance through the single graphene sheet was almost perfect, save a small reduction due to the reflection by the SiO_2_–air interface. The addition of the graphene sheet to the gold nanoantenna array blueshifted the transmission minimum from 4.10 μm to 3.63 μm. This blueshift of approximately −470 nm is in close agreement with the experimental observations (Table 1). Note that due to the simplified wavelength-independent dielectric coefficients of the theoretical models, additional vibrational mode features of SiO_2_ and Si substrate materials appearing in the experimental spectra from (2–10 µm) are not displayed in the simulated spectra.

## 4. Discussion

Similar blueshifts, of around −500 nm (~10%), were observed both from (i) direct comparison by graphene transfer onto pre-characterised metal metasurfaces and (ii) indirect comparison by patterning identical metal metasurfaces on graphene-covered- or bare- SiO_2_ substrates. A weaker blueshift, but much enhanced reflectance was also observed for the second photoresponse peak λ_2_ due to the Si–O vibration mode in the SiO_2_ layer (Table 1).

For the application relevant PMMA encapsulated (200 nm) metal metasurfaces and hybrid-graphene devices (Figure 3), FTIR revealed only a limited additional absorption in the MWIR region of interest, with some sharp characteristic features around 5.8 μm, and at longer wavelengths. However, a substantial redshift of around 400 nm was observed of λ_1_ for these devices.

These effects can have a significant practical impact on the design and functionality of such devices, where the optical response may require precise tuning for suitable operational efficiency. For example, for the application of molecular sensing where the absorption wavelengths can be relatively narrow. The demonstrated influence of the graphene layer, with indications of the range of sensitivity and application, also suggests the possibility of a further dynamic tunability of the system by modifying the graphene properties by electrostatic or electrochemical gating [55]. For a molecular sensing mechanism by peak wavelength shifts, the sharpness of the metasurface-induced MIR peaks could be further sharpened by replacing the metal nanoantenna arrays with high-q dielectrics and by introducing chirality to the geometrical design [36].

### Empirical Metasurface Photoresponse Calculator for Reliable Precision Design

**Figure 6:**
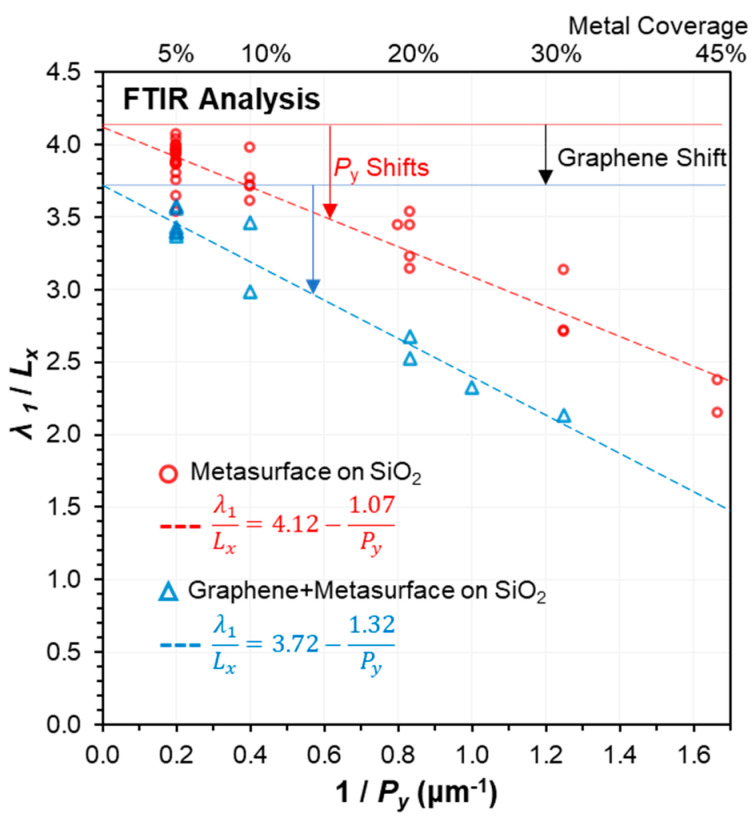
Dependence of the peak-response wavelengths on the metal length *L*x and lateral pitch *P*y. Strong blueshifts are evident for *P*y < λ_1_, and where the gold metasurfaces are integrated with graphene. Analysis of these trends enables simple and effective targeted design of the metasurface geometries.

The main factors determining the peak photoresponse wavelengths and interaction strength were experimentally observed to be the metal length *Lx*, the lateral pitch *Py*, and the material properties at the metasurface. From [56], we can expect the resonance condition for a single one dimensional metal antenna to occur at

(9) $$\frac{\lambda_{N}}{L_{x}+2\delta_{L}}=\frac{2n_{eff}}{{\;m}_{N}},$$

where *m_N_* is an integer denoting the order of the resonance condition, *n_eff_* is the effective mode refractive index, and *δ_L_* represents the deviation of the effective metal length from the geometrical length *L_x_*. Figure 6 shows the dependence of (*λ_N_*/*L_x_*) vs. (1/*P_y_*), revealing the overall trend for the metasurface arrays to be approximated by

(10) $$\frac{\lambda_{N}}{L_{x}}=A-\frac{b}{P_{y}},\ldots \delta_{L}=\frac{bL_{x}}{AP_{y}},$$

with extracted fitting parameters of *A*_(SiO2-Au)_ ≈ 4.12, *b*_(SiO2-Au)_ ≈ 1.07 for metasurfaces on SiO_2_ substrates. With the addition of graphene, we find *A*_(SiO2-Au-G)_ ≈ 3.72, *b*_(SiO2-Au-G)_ ≈ 1.32. From this, we can estimate *n*_eff(SiO2-Au)_ ≈ 2.06, and *n*_eff(SiO2-Au-G)_ ≈ 1.86. This pitch related shift effect can be quite substantial for subwavelength periodicities, e.g., for *P_y_* = 0.6 μm, ∆*λ*_1_ ≈ −40%.

The extraction of accurate fitting relations can further be used to improve the design precision of the metasurface geometries. This simple photoresponse design method was used to define the geometric parameters within this study to target specific wavelengths, for example in Figure 2 and Figure 3. Although we focused here on 4.25 μm, CO_2_, Table 2 indicates the expected design parameters to target a range of other MIR wavelengths and molecules.

The physics of the blueshift due to the insertion of the graphene sheet into the metal metasurface can be interpreted from two perspectives: (1) The electromagnetic wave is strongly perturbed, transiently, by the graphene sheet. This, in turn, affects the free electrons in the metal nanoantenna, resulting in a stronger plasmonic effect and a blueshift; (2) by viewing the graphene and the metal nanoantenna independently, the nanoantenna encloses a space in the form of a resonant cavity. The insertion of the graphene sheet reduces this cavity volume so that the resonant frequency is increased, resulting in a blueshift. Since this affect is expected to be influenced by the graphene charge carrier density, it is anticipated that the spectral photoresponse wavelength of the hybrid graphene metasurfaces can be further tuned or modulated by electrostatic or electrochemical gating [38,39,40].

## 5. Conclusions

In summary, we demonstrated simple, precise, and reproducible geometrical photoresponse tuning of hybrid graphene metasurface towards targeted molecular sensing. Systematic midinfrared photoresponse characterisation was enabled by a combination of electron beam lithography-based nanofabrication, micro-FTIR spectroscopy, and FDTD studies. Peak-response wavelengths were found to depend most critically on two geometrical parameters; the longitudinal metal nanoantenna length and the lateral pitch between the antenna. Substantial blueshifts were observed and characterised upon the integration of graphene with the metal metasurfaces, and for high density metasurface structures, observed up to ~40%. The careful interpretation of more than 100 unique structures enabled the development of a simple and precise set of design tools to ensure geometrically fine-tuned photoresponses for reproducible mid-infrared molecular targeting. The combination of hybrid metasurfaces and their detailed characterisation is important for the next generation of smart portable sensors and lab-on-chip technologies.

## Figures and Tables

**Table 1:** Graphene-induced blueshifts in the FTIR peak photoresponses of metasurface arrays following transfer of monolayer CVD graphene (G).

Identification		Metal Geometry, μm				Peak Photoresponse Wavelengths, μm					
Sample	Position	P_x_	P_y_	L_x_	L_y_	λ_1_	λ_1_ (+G)	∆λ_1_ (G)	λ_2_	Λ_2_ (+G)	∆λ_2_ (G)
FDTD	Simulation	1.60	1.2	1.4	0.2	4.10	3.63	−0.47	-	-	-
G1	A	1.40	2.5	1.18	0.24	4.02	3.81	−0.21	9.71	9.30	−0.41
G1	B	1.30	5.0	1.18	0.24	4.26	3.94	−0.32	9.78	9.40	−0.38
G2	A	1.65	5.0	1.54	0.30	5.10	4.72	−0.38	9.90	9.75	−0.15
G2	B	1.65	5.0	1.53	0.31	5.45	4.75	−0.70	9.92	9.79	−0.13
G2	C	2.40	5.0	1.54	0.31	5.70	4.80	−0.90	9.94	9.82	−0.12
Average						4.91	4.40	−0.50	9.85	9.61	−0.24

**Table 2:** Estimated metal metasurface geometries for midinfrared molecular targeting, for a range of metal lengths *L*_x_ and lateral pitch *P*_y_, with and without the presence of graphene.

			CH_4_	CO_2_	N_2_O	CO	O_3_	NO
Material	*P_y_* (μm)	δ_L_ (%)	*L_x_* (μm)					
Au-SiO_2_	10	3	0.82	1.06	1.12	1.16	1.18	1.30
	5	5	0.84	1.09	1.15	1.20	1.21	1.33
	2.5	10	0.89	1.15	1.22	1.26	1.28	1.41
	1.2	22	1.02	1.32	1.39	1.45	1.47	1.61
G-Au-SiO_2_	10	4	0.92	1.18	1.25	1.30	1.32	1.45
	5	7	0.95	1.23	1.30	1.35	1.37	1.50
	2.5	14	1.03	1.33	1.41	1.46	1.48	1.63
	1.2	30	1.26	1.62	1.72	1.78	1.81	1.98
**λ (μm)**			**3.3**	**4.25**	**4.5**	**4.67**	**4.74**	**5.2**

## Data Availability

The data presented in this study are available on request from the corresponding authors.
